# MiR-146/TNF-α/IL-6/osteocalcin crosstalk in anti-rheumatic potential of *Galleria mellonella* hemolymph from computational molecular modeling to in-vivo validation

**DOI:** 10.1007/s10822-025-00595-3

**Published:** 2025-04-23

**Authors:** Sara M. Ahmed, Elham Ali, Amina R. Ali, Mona A. Mohamed, Hemmat Mansour Abdelhafez, Alya Mashaal

**Affiliations:** 1 Special Zoology, Zoology and Entomology Department, Faculty of Science (for Girls), Al-Azhar University, Cairo, Egypt; 2 Molecular Biology, Zoology and Entomology Department, Faculty of Science (for Girls), Al-Azhar University, Cairo, Egypt; 3 Environmental Health, Pesticide Chemistry Department, National Research Centre, Giza, Egypt; 4 Biochemistry Division, Chemistry Department, Faculty of Science (for Girls), Al-Azhar University, Cairo, Egypt; 5 Cytochemistry and Histology, Zoology and Entomology Department, Faculty of Science (for Girls), Al-Azhar University, Cairo, Egypt; 6 Immunology, Zoology and Entomology Department, Faculty of Science (for Girls), Al-Azhar University, Cairo, Egypt

**Keywords:** Rheumatoid Arthritis, *G. mellonella* hemolymph, MiR-146a, Immunomodulators, Osteocalcin, In-silico simulation

## Abstract

Rheumatoid arthritis (RA) is an inflammatory autoimmune illness that persistently and recurrently affects joints. In RA, miR-146a functions as a key regulator, modulating inflammation by targeting and downregulating cytokines that promote inflammation, such as TNF-α and IL-6, whereas osteocalcin, a bone metabolism marker, plays a role in bone remodeling and joint health. The interplay between these molecules significantly influences RA progression and severity by balancing inflammation and bone integrity. Conventional antirheumatic drugs often cause varying levels of side effects. As sustainable development initiatives grow, insects are gaining interest as sustainable food sources and potential medicinal agents. Notably, the increasing *Galleria mellonella* (*G. mellonella*) population has raised concerns about the spread of honeybee viruses, affects bee products and food security, and drives economic losses in the therapeutic market. Accordingly, hemolymph has crucial defensive and immunological effects in insects and has recently been investigated as an immunomodulatory agent in parasitic in-vitro and in-vivo rat models. This work was designed to elucidate the potential immunomodulatory impact of *G. mellonella* hemolymph on the crosstalk between miR-146a, IL-6, TNF-α, and osteocalcin in the context of RA, utilizing both computational molecular modeling and in-vivo validation. Computer-aided molecular simulation for immune and RA mediators is applied through specific cell annotation, targeted pathways, and in-silico protein‒protein and gene‒gene interactions with a gene relative-tissue expression heatmap, which is based on gas chromatographic‒mass spectrometric analysis of hemolymph. Our study is the first to adapt a preliminary test to optimize hemolymph dosing and toxicity. The rats were subsequently divided into four groups: healthy control, Freund’s adjuvant-induced arthritis (utilized as a model that mimics human RA), methotrexate-treated arthritis, and hemolymph-treated arthritis groups. Our findings indicate that hemolymph contains valuable active compounds that have anti-inflammatory and antioxidant potential, increasing the impact of recovery on diseased joints in comparison with the arthritic and methotrexate groups. This is the first report investigating the maximum inhibition rate of *G. mellonella* hemolymph as an immunomodulatory and anti-inflammatory agent in an arthritic model.

**Graphical abstract:**

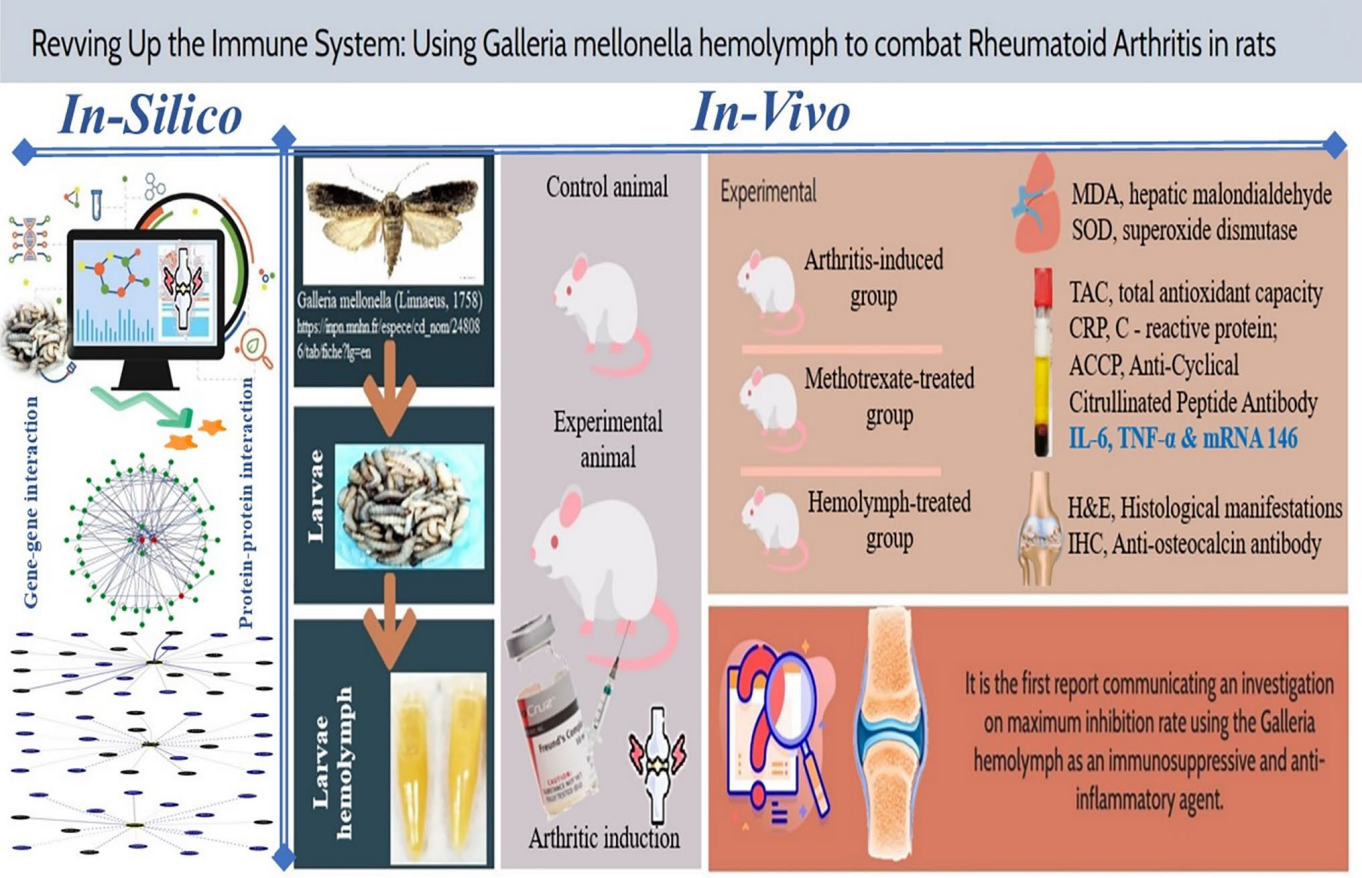

**Supplementary Information:**

The online version contains supplementary material available at 10.1007/s10822-025-00595-3.

## Introduction

Rheumatoid arthritis (RA) is a progressive autoimmune condition characterized by inflammation of the synovial membrane, deterioration of bone and cartilage, and involvement of extra-articular tissues. The increasing age-standardized prevalence and incidence of RA make it a significant global public health concern [1]. Women are more frequently affected by RA compared to men [2]. The disease triggers the production of several antigens, which activate inflammatory mediators [3], highlighting the need for effective therapeutic strategies targeting these mediators.

Current RA treatments focus on reducing inflammation, relieving discomfort, and limiting joint damage. This is achieved through a combination of analgesics, disease-modifying antirheumatic drugs (DMARDs), glucocorticoids, nonsteroidal anti-inflammatory drugs (NSAIDs), and specific mediator response inhibitors [1]. Methotrexate (MTX) is the first-line DMARD for RA treatment due to its efficacy and cost-effectiveness. However, approximately 30% of patients experience an insufficient response to MTX, and many discontinue its use due to toxicity [4]. Additionally, MTX is associated with several undesirable side effects, including gastroenteropathy, immunosuppression, secondary tumor growth, infection, and organ damage. Therefore, there is an urgent need for novel, naturally derived antirheumatic agents with improved pharmacological properties [5].

*Galleria mellonella*, commonly known as the greater wax moth, is an insect from the *Pyralidae* family in the *Lepidoptera* order. It is known for parasitizing honeybees and their hives. *G. mellonella* larvae consume pollen, the wax comb's midrib, bee larvae cast skins, and trace amounts of propolis and honey. The silk-lined tunnels they create can trap and starve emerging bees, leading to significant comb destruction, a condition known as galleriasis [6]. Beyond its ecological role, *G. mellonella* has gained recognition as an experimental model organism due to its immune system's similarity to that of mammals [7].

*G. mellonella* hemolymph is rich in antimicrobial, antibacterial, antifungal, and antiviral activities and exhibits anticancer properties due to its defense peptides with diverse biochemical properties [8]. Given its strong immunomodulatory potential, hemolymph could be explored as a natural anti-inflammatory and antirheumatic agent. This is particularly relevant to inflammatory diseases such as RA, where microRNA-146a (miR-146a) plays a crucial regulatory role.

MiR-146a has emerged as a negative feedback regulator of autoimmunity and the inflammatory response. It modulates the IRAK-1/TRAF6/NF-κB pathway, downregulating IRAK-1 and TRAF6 expression, thereby inhibiting NF-κB activation and reducing proinflammatory mediator production [9]. This regulatory function modulates the expression of inflammatory genes [10], and  makes miR-146a a critical player in RA pathogenesis [11]. MiR-146a, interleukin-6 (IL-6), tumor necrosis factor-alpha (TNF-α), and osteocalcin (OST) interact in complex networks within inflammatory pathways, influencing disease progression [12].

IL-6 acts as a proinflammatory cytokine, promoting the synthesis of acute-phase proteins and stimulating inflammatory processes. Aberrant IL-6 expression is strongly associated with chronic inflammation and autoimmune disorder pathogenesis. In RA, IL-6 drives systemic inflammation and promotes sustained inflammatory responses within joint fluid, distinguishing RA from osteoarthritis [13]. TNF-α triggers inflammation and tissue destruction by activating inflammatory signaling pathways and influencing IL-6 production. RA progresses through three stages: an initial nonspecific inflammatory phase, amplified by T-cell activation in the synovium; a chronic inflammatory stage; and a tissue damage stage driven by cytokines such as IL-1, IL-6, and TNF-α [14]. These cytokines collectively amplify the inflammatory response, worsening RA pathology.

Osteocalcin, a bone matrix protein involved in bone metabolism, plays a crucial role in modulating the interconnection between the inflammatory process and bone remodeling [11]. This interconnection underscores the complexity of RA pathophysiology. Given this complexity, identifying novel therapeutic agents that target these interactions is essential. The exploration of *G. mellonella* hemolymph spans computational molecular modeling through to in-vivo validation, providing a comprehensive approach to understanding its potential as a therapeutic agent.

Therefore, the objective of this study was to investigate the immune-modality impact of *G. mellonella* hemolymph, as potential anti-inflammatory and therapeutic antirheumatic natural agent, on the crosstalk between miR-146a, IL-6, TNF-α, and osteocalcin in comparison with MTX in adjuvant-induced arthritis (AIA), spanning computational molecular modeling through to in-vivo validation.

## Materials and methods

### Ethics committee approval

All research methods adhered to a well-established ethical framework in accordance with the study design. Ethical approval was granted by the Research Ethics Committee of the Faculty of Medicine (for Girls), Al-Azhar University, Cairo, Egypt (Reference No. 202305/924). The study also complied with the principles outlined in the National Institutes of Health’s Guide for the Care and Use of Laboratory Animals (NIH Publication No. 85-23, revised 1996) and adhered to the ARRIVE 2.0 guidelines, ensuring transparency and rigorous reporting standards.

### Preparation of *G. mellonella* larvae hemolymph extract

The larvae of *G. mellonella* were cultured in the animal house of the Zoology and Entomology Department, Faculty of Science (Girls’ Branch), Al-Azhar University. They were reared on an artificial diet prepared following the method of Wright et al. [15]. Hemolymph was collected from 5th instar larvae using the technique described by Lee et al. [16].

### Preliminary test and hemolymph analysis and dosing

The experimental procedure, high-performance liquid chromatography (HPLC) analysis of active compounds, and dosage selection were based on preliminary experiments. The potential toxic effects of *G. mellonella* larval hemolymph extract were assessed by determining the LD50. This test was conducted in accordance with OECD-423 guidelines [17] to minimize the number of experimental animals and establish the optimal hemolymph dose. Hemolymph was administered orally to rats as a single dose at varying concentrations (1000, 2000, 3000, 4000, 5000, 7000, and 10,000 mg/kg body weight). The treated rats were then monitored for 14 days post-administration.

### In-silico application

In-silico analysis provides a crucial foundation for understanding the molecular mechanisms underlying RA and supports the in-vivo investigation of the inflammatory cytokines miR-146a and OC.

#### PICKLE (knowledgebasE protein interaction)

PICKLE (Protein InteraCtion KnowLedgebasE), University of Patras, Greece. Release 3.3, 1 October 2021. http://www.pickle.gr/ (Accessed on 3 March 2024) was used to predict CRP and osteocalcin interactions in relation to PADI4 (Id: NC_000001.11:g.17157671G > T; Variation ID: 590,889; Accession: VCV000590889.1), a common variant gene of arthritis (ClinVar, https://www.ncbi.nlm.nih.gov/clinvar/?term=arthritis). PICKLE serves as a meta-database designed specifically for the direct protein‒protein interactome, seamlessly integrating various publicly available protein‒protein interaction (PPI) databases through genetic information ontology. The visualization aspect uses Cytoscape.js 3.3.0.

#### Analyzing and visualizing gene expression

Analysis and visualization of BGLAP, IL-6, TNF-α, and PADI4 gene expression in bone- and adipose stromal-specific tissues related to arthritic induction were carried out via the scRNA-seq database HUSCH, accessed November 24, 2024 (http://husch.comp-genomics.org/#/search), and detailed cell-type annotations were provided on the basis of the curated reference.

#### Gene–gene pathways and interactions by bioinformatics analysis

Prediction of the top interacting genes for CRP, OST (BGLAP, bone gamma-carboxyglutamate protein, Gene ID: 632, https://www.ncbi.nlm.nih.gov/gene/632), and PADI4 was performed through gene interaction analysis at the University of California Santa Cruz (UCSC) Genome Browser RRID: SCR_005780. The UCSC Genomics Institute website is http://genome.ucsc.edu/index.html (Accessed on 3 March 2024).

### Experimental animals

Twenty-five mature male *Rattus rattus* (albino rats), each weighing approximately 110 ± 10 g, were used in this study. The animals were obtained from the National Research Centre (NRC) animal house in Dokki, Cairo, Egypt. Before the experimental procedures, they were acclimatized for two weeks in sanitized, specially designed cages (five rats per cage) under controlled conditions, including a 12-h light/dark cycle, optimal temperature, and adequate ventilation. Throughout the study, the rats were provided with a standard rodent diet and water ad libitum.

### Induction of arthritis

Complete Freund’s adjuvant (CFA) was purchased from Santa Cruz Biotechnology (sc-24018, Lot # K1620), INC, Delaware Avenue, CA 95060-5706, USA. The adjuvant-induced arthritis (AIA) model was induced by a single sub-plantar injection of CFA (100 μL/animal) into the left hind paw, as described previously [18]. Following the CFA injection, the animals exhibited noticeable edema within 24 h and developed significant joint inflammation and arthritis by day 14. Arthritis was confirmed by positive rheumatoid factor and antinuclear antibody tests (not recorded).

### Experimental design

Twenty male* Rattus rats* were randomly grouped into the following; a control healthy group (C), which received distilled water (1 ml) orally daily; an arthritis-induced group (RA); a methotrexate-treated arthritis group (2 mg/kg weekly by oral gavage according to [19] (RA + MTX)); and a hemolymph-treated arthritis group (300 mg/kg b.wt *G. mellonella* hemolymph extract daily by oral gavage; according to the LD_50_ test) (RA + GHE). The treatment regimen lasted for 14 days after CFA-induced arthritis induction. The initial and final body weights were recorded to demonstrate the body weight gain in all the experimental groups.

### Blood collection and tissue sampling

On the 30th day, the rats were anesthetized with 1.5% isoflurane, blood samples were drawn from the retro-orbital plexus and centrifuged at 4000 rpm for 5 min, and the serum was stored at −20 °C. After being sacrificed by cervical dislocation, the rats were dissected to obtain the relative organ weights of the liver, kidney, spleen and heart in the different groups.

In addition, joint samples were collected and processed for further examination. The liver was removed quickly and perfused with PBS (pH 7.4) to eliminate any blood clots. The liver homogenate was prepared by homogenizing 0.5 g of hepatic tissue via an automatic homogenizer (RAT-MICCRA D-S, MICCRA GmbH, Heitersheim, Germany) in 5 mL of PBS (pH 7.4) to produce a 10% (w/v) hepatic homogenate. The homogenate samples were then centrifuged at 4000 rpm for 15 min at 4 °C, and the supernatant was collected for the assessment of malondialdehyde (MDA) content.

### Assessment of inflammatory infiltration

The severity of arthritis was assessed by measuring inflammatory cell infiltration through paw edema. Paw swelling was quantified using digital electronic calipers [20], with changes over time indicating the degree of inflammation. The inflamed paw was compared to the contralateral non-inflamed paw or baseline measurements taken before arthritis induction. A collection of manually drawn pathway maps showing that abnormal activation of the immune system modulates proinflammatory cytokine and chemokine levels, promotes synovial angiogenesis and promotes leukocyte infiltration was generated via KEGG PATHWAY (https://www.genome.jp/pathway/hsa05323).

### Hematological and biochemical assays

The CBC parameters were measured via an automated hematological analyzer (XN–1000–Hematology–Analyzer–Sysmex Corporation, Japan). The values for white blood cells (WBCs), red blood cells (RBCs), hemoglobin (Hb), and platelets (PLTs) were derived from the CBC data.

Serum alanine aminotransferase (ALT), aspartate aminotransferase (AST) and alkaline phosphatase (ALP) levels were assayed via commercial kits (Spectrum, Germany). Serum creatinine and urea were assayed via the colorimetric method via commercial kits (Biodiagnostic, Egypt).

### Assessment of oxidative biomarkers

The oxidative response was assayed via a commercial diagnostic kit for total serum antioxidant capacity (TAC; CAT# STA-360, Biodiagnostic, Egypt). The concentrations of hepatic MDA, a product of lipid peroxidation (MDA; CAT# NWK-MDA01 Biodiagnostic, Egypt), and superoxide dismutase (SOD; CAT#SD2521 Biodiagnostic, Egypt) were also estimated.

### Assessment of inflammatory mediators

The inflammatory response was assayed via Quantikine ELISA kits to detect the following diagnostic inflammatory markers in the serum: the anti-cyclical citrullinated peptide antibody (ACCP) level (MyBiosource; Rat ACCP Quantikine ELISA Kit, Catalog # MBS2606863, USA) and the C-reactive protein (CRP) level (MyBiosource; Rat CRP Quantikine ELISA Kit, Catalog # MBS2508830, USA). The interleukin-6 (IL-6) level was measured via the Rat IL-6 Quantikine ELISA Kit (Catalog # SR6000B, R&D Systems). Tumor necrosis factor-α (TNF‐α) levels were determined via a Rat TNF‐α Quantikine ELISA Kit (catalog # CSB-E11987r, Cusabio).

### Real-time PCR

To study the expression level of miR-146a among the research groups, following the manufacturer's instructions, serum samples were treated with the mirVana™ miRNA Isolation Kit (Cat. No. AM1560, Ambion) to extract total RNA. The extracted RNA was subsequently reverse transcribed into cDNA via MultiScribe™ Reverse Transcriptase with the TaqMan™ MicroRNA Reverse Transcription Kit (Cat. No. 4366597; Applied Biosystems). The miR-146a expression level was detected via a TaqMan™ MicroRNA Assay (Cat. No. 4440888) on a step-one real-time PCR system (Applied Biosystems) and normalized to that of small nuclear RNA (U6). The sequences of primers used were as follows: for miR-146a, F: TGAGAACTGAATTCCATGGGTT. The reverse sequence was the universal adaptor PCR primer in the kit. For RNU6: F: CTCGCTTCGGCAGCACA and R: AACGCTTCACGAATTTGCGT. The data were quantified via the 2^−ΔΔCT^ method [21].

### Histopathological and immunohistochemical techniques

Each group of animals’ left hind paws were peeled, fixed in formalin for 48 h, and then decalcified for one month in 15% EDTA. Following decalcification, the tissues were embedded in paraffin and cut into 4 μm-thick sections. The pathological alterations were then revealed by processing and staining the sections with hematoxylin and eosin (H&E) [22] and anti-OST, which are markers of mature osteoblasts and osteoarthritic chondrocytes detected in the joint tissue structure.

For image analysis, an Olympus digital camera (Olympus LC20-Japan) mounted on an Olympus microscope (Olympus BX-50, Japan) was used to digitize the slides. Using Video Test Morphology 5.2 software (Russia) with a dedicated built-in procedure for immunohistostaining analysis and stain quantification, the resulting images were examined on an Intel® Core I3®-based computer. For the purpose of evaluating positive cells via image analysis software (JID801D), three fields per slice were selected at random.

### Statistical analysis

Statistical analyses of variance were performed via post hoc tests with ANOVA in accordance with the methodology of Ridgman [23]. The SPSS program (Statistical Analysis for Social Science, Version 23) was used to process and analyze the data. P < 0.05 was regarded as statistically significant, and the data are displayed as the means ± SDs.

## Results

### Hemolymph analysis and dose optimization

HPLC analysis of *G. mellonella* hemolymph provided insights into the presence and quantification of various bioactive compounds with antioxidative and anti-inflammatory properties. These include quinic acid, ellagic acid, cinnamic chlorogenic acid and resorcinol, enanthic acid, butyric acid, pentadecanoic acid, myristoleic acid, heneicosanoic acid, arachidonic acid, stearic acid, oleic acid, linoleic acid, eicosatrienoic acid, and eicosapentaenoic acid.

**Fig. 1:**
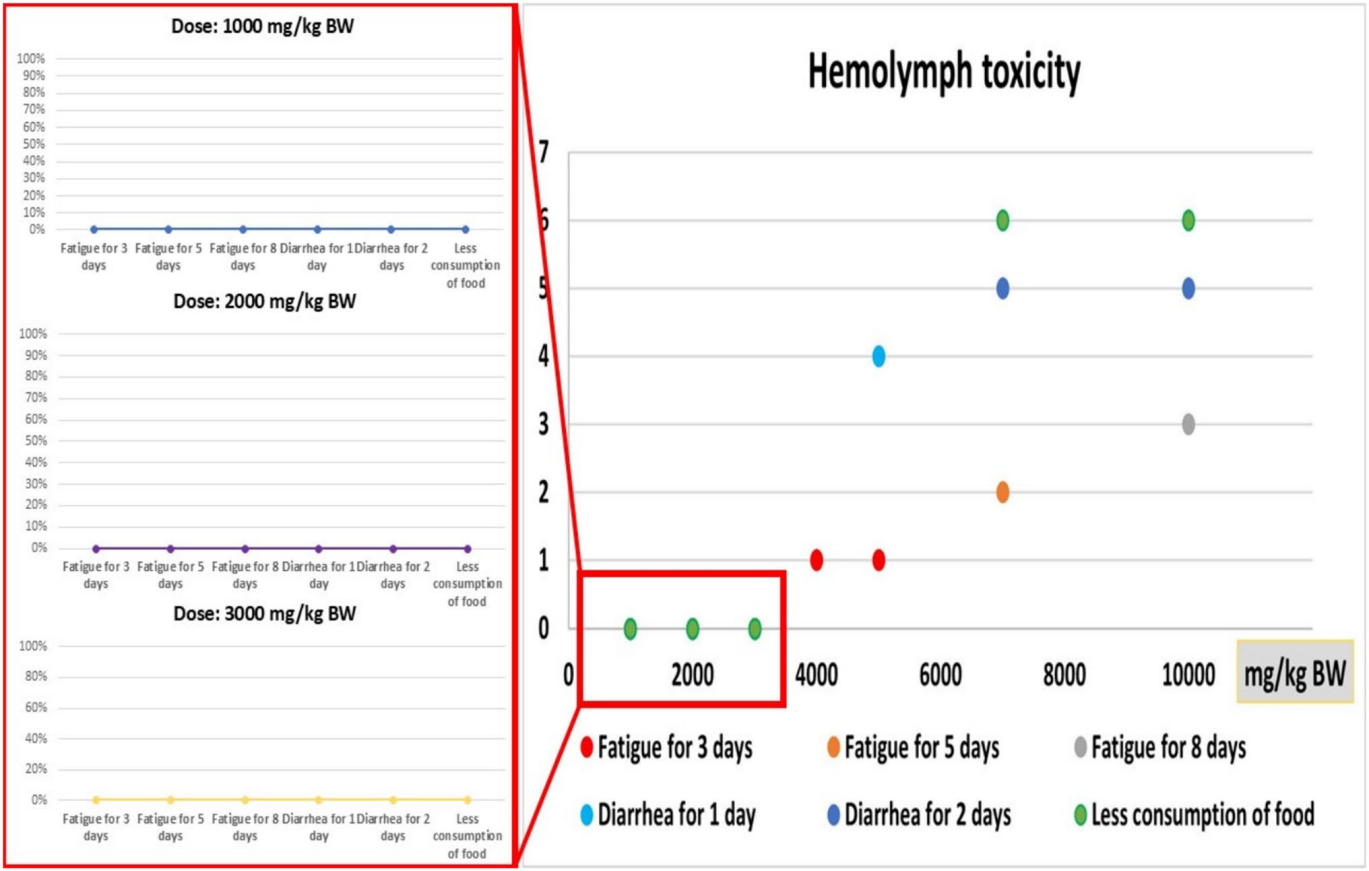
Acute toxicity of hemolymph illustrated the correlation between administrated doses (mg/kg BW) and toxicity signs

The hemolymph was found to have a very low level of toxicity and did not cause any mortality exceeding 10,000 mg/kg body weight. On the basis of the acute toxicity studies shown in Fig. 1, the LD_50_ of the hemolymph is estimated to be greater than 3000 mg/kg b wt (highest concentration without any signs). An acceptable daily intake (ADI) dose of 300 mg/kg b wt was estimated (1/10 LD_50_) and applied to test its effect on arthritis-induced rats.

### PICKLE/HUSCH database prediction

**Fig. 2:**
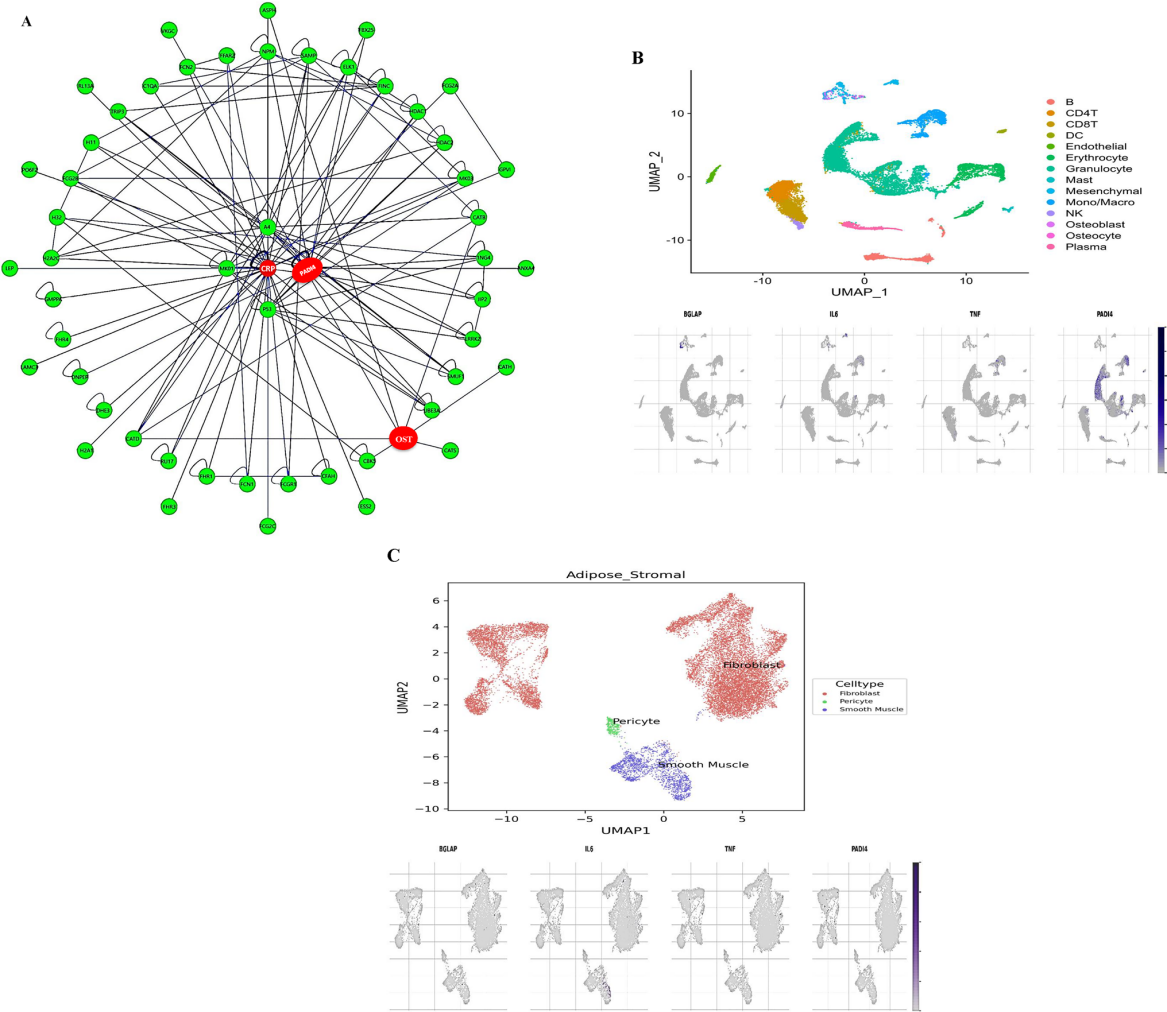
**A** Concentric layout network visualization of CRP/OST/PADI4 interaction. Data retrieved from PICKLE (Accessed on 3 March 2024). The concentric layout displays 54 nodes (proteins) and 139 edges (interactions), with red nodes highlighting the central proteins of interest (CRP, OST, and PADI4) and green nodes representing first-neighbor interacting proteins. The edges depict known protein–protein interactions, derived from high-quality experimental and computational sources (PPI quality level 2). Self-loops indicate proteins with autointeractions. The network was normalized at the protein level (UniProt) and filtered using the default cross-checking method. The network structure reveals highly interconnected hub proteins, which may indicate potential regulatory roles in signaling pathways. Highly interconnected nodes, suggests functional relationships and co-regulation of biological pathways; Self-looping proteins, indicate proteins with autointeractions or self-regulatory functions; Cross-talk between pathways, CRP, OST, and PADI4 may bridge different biological processes, influencing inflammation, immune response, and protein modifications. This network can help identify potential therapeutic targets and guide further studies into inflammatory disease mechanisms. **B** Bone tissue specific cells annotation, data retrieved from HUSCH, comparative gene exploration for BGLAP, IL-6, TNF-α, and PADI4 expression in bone tissue. **C** Adipose stromal specific cells annotation, data retrieved from HUSCH, comparative gene exploration for BGLAP, IL-6, TNF-α, and PADI4 expression in adipose stromal tissue

Figure 2A displays the CRP/OST/PADI4 interactions that were explored with other genes and with each other. This information was obtained from the PICKLE database. The interaction between CRP, OST, and arthritis, specifically involving PADI4 (Peptidyl Arginine Deiminase 4), is an area of research focused on understanding the pathogenesis of arthritis. Hence, CRP is an acute-phase protein associated with inflammation, OST, a bone-derived protein that particularly plays a key role in bone remodeling processes related to arthritis, and PADI4, an enzyme involved in the posttranslational modification of proteins by citrullination, has been linked to autoimmune responses in arthritis, as citrullinated proteins are often targeted by the immune system in rheumatoid arthritis. The current in-silico simulation of their interaction revealed complex and ongoing crosstalk mechanisms and pathways that modulate the development and progression of arthritis. By predicting the interactions of CRP and OST with PADI4, a key arthritis-associated gene, PICKLE helps elucidate how these proteins contribute to inflammatory pathways. This finding directly correlates with the in-vivo evaluation of osteocalcin’s role in joint inflammation and bone remodeling.

Figures 2B & C present cell annotations and an examination of the expression of BGLAP (osteocalcin), IL-6, TNF-α, and PADI4 in arthritis-relevant tissues, which provide insights into their involvement in RA pathophysiology. This finding aligns with the in-vivo assessment of cytokine levels and miR-146a regulation, reinforcing the molecular link between systemic inflammation and bone metabolism.

Annotation of cell types in bone tissue (dataset: HU_0370_Bone_GSE169396) is shown in Fig. 2B. The breakdown of observational BGLAP (osteocalcin), IL-6, PADI4, and TNF-α expression levels revealed high expression of IL-6, TNF-α, and PADI4 in monocytes/macrophages, supporting their central role in driving inflammation and immune activation in arthritis. These cytokines contribute to osteoclast activation, leading to bone resorption. The exclusive expression of BGLAP in osteoblasts/osteocytes highlights its involvement in bone matrix production and integrity, which may be disrupted by inflammatory cytokines such as IL-6 and TNF-α.

Annotation of cell types in adipose stromal tissues is shown in Fig. 2C. The chart highlights the expression patterns of BGLAP, IL-6, PADI4, and TNF-α. Elevated expression of IL-6 and TNF-α in pericytes and smooth muscle cells indicates their involvement in inflammatory signaling and tissue remodeling within adipose stromal tissues, which may contribute to arthritis-associated inflammation. Minimal or negligible expressions of BGLAP suggested a limited role in adipose stromal tissues compared with bone-related contexts.

### Gene–gene interactions and pathways

**Fig. 3:**
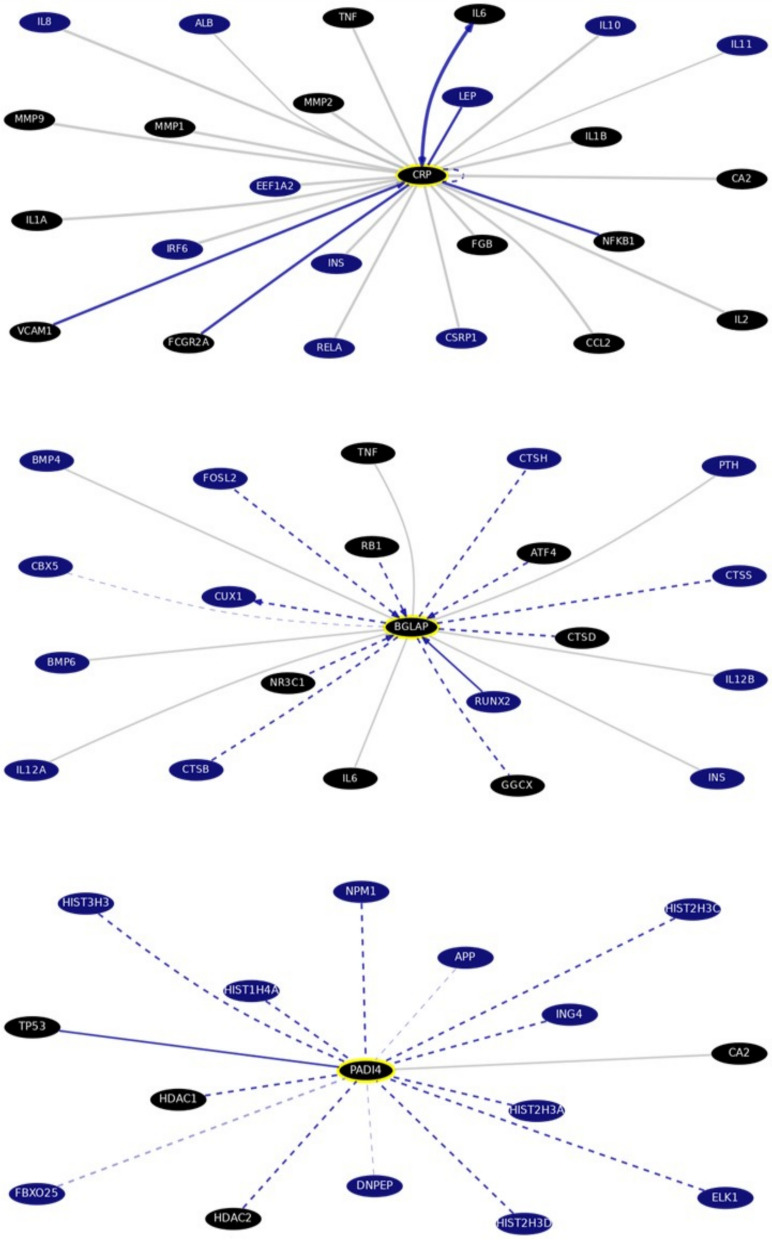
CRP/OST(BGLAP)/PADI4 top interacting genes. Data retrieved from UCSC Genome Browser Gene Interaction Graph, highlighting the Drug Bank interaction, obtained via gene interactions and pathways. Accessed on 3 March 2024. CRP: chr1:159682078–159684379; BGLAP: chr1:156211950–156213123; PADI4: chr1:17634689–17690495. Interactions are colored by support: Black colored genes: treatment hits by Drug Bank; Grey: interaction from several datasets with only text mining; light blue: interaction database; blue: pathway database; Dashed lines indicate interactions without text mining support

The CRP, OST (BGLAP), and PADI4 gene‒gene interactions and pathways, as compiled from curated databases and text mining at the UCSC Genomics Institute, are shown in Fig. 3. The interactions and pathways involving these genes are of interest for understanding various biological processes, particularly in the context of arthritis as an inflammatory disease. Figure 3 provides a brief overview of these interactions and pathways, which are likely complex and multifaceted, contributing to various aspects of inflammation, bone metabolism, and autoimmune responses involved in arthritis. Identifying the top interacting genes for CRP, BGLAP, and PADI4 allows for a deeper understanding of their regulatory networks. This bioinformatics analysis supports the in-vivo exploration of how miR-146a modulates inflammatory signaling pathways, particularly the IL-6 and TNF-α pathways, in RA progression. Studying these interactions and pathways can provide insights into disease mechanisms and potential therapeutic targets for managing inflammatory and autoimmune conditions.

These in-silico predictions are corroborated by in-vivo results, offering valuable insights into the molecular mechanisms underlying RA pathogenesis and the potential therapeutic effects of *G. mellonella* hemolymph. This integrative approach, encompassing computational molecular modeling and in-vivo validation, underscores the significance of these interactions in the development of novel antirheumatic treatments.

### Body and relative organ weights

Compared with control rats, rheumatoid arthritis and RA + MTX rats presented highly significant reductions (p < 0.01) in terms of body weight gain (Table 1). Compared with control rats, hemolymph arthritis-treated rats presented a slight decrease in body weight gain that was not statistically significant but was significantly greater (p < 0.01) than that of the RA + MTX group.

**Table 1:** The body and relative organ weight

Groups	C	RA	RA + M	RA + GHE
Parameter				
Relative Organs Weight (g) ± SD				
Liver	4.05 ± 0.45	4.46 ± 0.74	4.30 ± 0.56	4.75 ± 0.18^a^
Kidneys	0.70 ± 0.13	0.72 ± 0.02	0.70 ± 0.14	0.65 ± 0.03
Heart	0.34 ± 0.03	0.40 ± 0.06	0.45 ± 0.21	0.36 ± 0.03
Spleen	0.51 ± 0.10	0.48 ± 0.25	0.61 ± 0.13	0.62 ± 0.06
Mean Body weight gain (g) ± SD	145.20 ± 19.73	110.20 ± 20.94^a^	106.20 ± 25.34^a^	139.60 ± 6.11^c^

Each value represented the mean ± standard deviation (SD); n = 5 rats. *C* normal control; *RA* Rheumatoid Arthritis; *M* Methotrexate; *GHE* hemolymph. The values are considered significant at p < 0.05

^a^significance versus C

^b^significance versus RA

^c^significance versus RA + M

The liver, kidney, and heart relative weights of the RA and RA + MTX rats were not significantly greater than those of the control group. The same result was observed for the relative weight of the spleen in the RA + MTX group. The relative spleen weight in the arthritic group was not significantly lower (p < 0.05) than that in the control group (Table 1).

Similarly, when hemolymph extract was administered, the relative liver weight dramatically increased (p < 0.05). Additionally, a nonsignificant increase in the heart and spleen relative weights and a nonsignificant reduction in the kidney relative weight were observed relative to those in the control group (Table 1).

### Inflammatory infiltrates

**Fig. 4:**
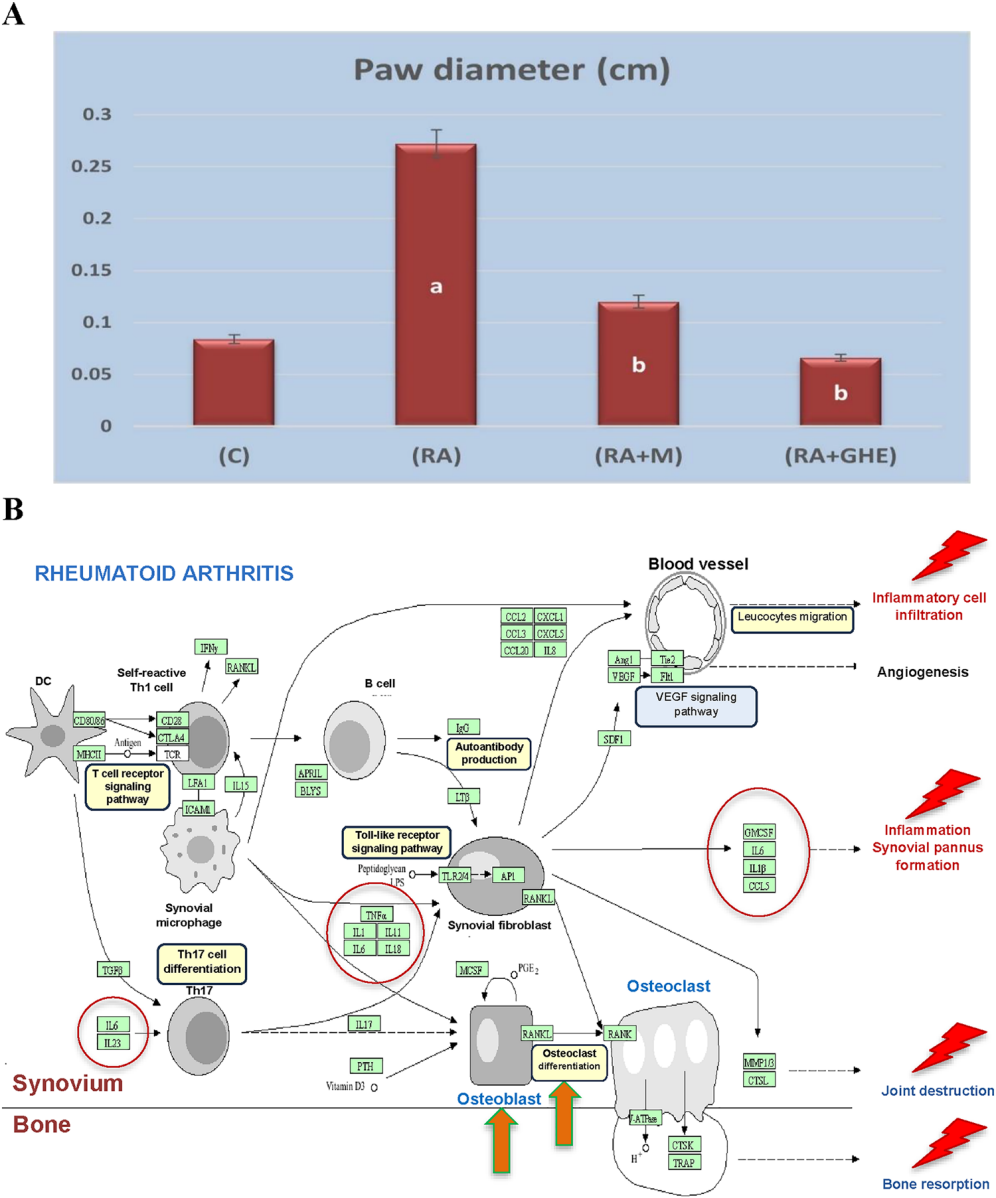
**A** Change in the paw diameter (cm) represented as (mean ± SE) in different experimental groups. Control group (C), Rheumatoid arthritis group (RA), Methotrexate arthritic treated group (RA + M), and hemolymph arthritic treated group (RA + GHE). The mean difference is significant at p < 0.05. a: significant vs control, b: significant vs RA group. **B** Drawn pathway maps of synovial angiogenesis and leukocyte infiltration, obtained via KEGG PATHWAY Database, accessed 25 November 2024

Figure 4A shows the paw diameter change (cm) in the different studied groups as an indicator of inflammatory infiltration, which is typically perivascular expression. Edema in the arthritic paw refers to the accumulation of inflammatory cells within the paw, accompanied by edema or swelling. Compared with the control group (0.0840 ± 0.01), the rheumatoid arthritis group (RA) presented a highly significant increase (p < 0.01) in the mean paw diameter (0.272 ± 0.08), whereas both the methotrexate (RA + MTX)- and hemolymph-treated groups (RA + GHE) presented nonsignificant changes (0.120 ± 0.04), whereas the *G. mellonella* hemolymph-Arthritis group (RA + GHE) presented a nonsignificant decrease (0.066 ± 0.01) in paw diameter. These results suggest that *G. mellonella* hemolymph may have a mitigating effect on arthritis comparable to that of MTX.

Abnormal activation of the immune system elevates proinflammatory cytokine levels, which can promote synovial angiogenesis and leukocyte infiltration. The synovium forms a hyperplastic pannus with infiltrated macrophage-like and fibroblast-like synoviocytes and invades joints by secreting proteinases and inducing osteoclast differentiation. These data underscore the cell-specific expression of inflammatory mediators and bone-related markers, offering insights into tissue-specific regulatory mechanisms in arthritis (Fig. 4B).

### Hematological and biochemical findings

Table 2 shows the hematological and biochemical changes. Compared with those in the control group, the average Hb and PLT values were significantly lower (p < 0.001) and RBC counts (p < 0.005) in the RA group. However, the WBC value highly significantly increased (P < 0.001).

**Table 2:** Hematological and biochemical modulators

Groups	C	RA	RA + M	RA + GHE
*Parameter*				
HG (g/dL)	13.28 ± 0.47	11.62 ± 0.88^a^	12.54 ± 0.46^b^	13.10 ± 0.34^b^
RBCs (*10^12^/L)	7.94 ± 0.18	7.13 ± 0.63^a^	7.26 ± 0.51^a^	7.78 ± 0.41^b^
PLTs (*10^9^/L)	800.80 ± 42.91	652.60 ± 11.84^a^	597.20 ± 28.71^ab^	792.60 ± 9.89^bc^
WBCs (*10^9^/L)	8.68 ± 0.69	11.06 ± 0.95^a^	6.20 ± 0.48^ab^	8.70 ± 0.45^bc^
ALT (U/L)	28.52 ± 0.42	100.08 ± 0.55^a^	80.79 ± 1.60^ab^	42.37 ± 0.79^abc^
AST (U/L)	53.72 ± 1.91	99.10 ± 2.96^a^	86.02 ± 4.42^ab^	60.94 ± 2.26^abc^
ALP (U/L)	98.11 ± 3.86	429.29 ± 9.72^a^	302.99 ± 12.28^ab^	209.61 ± 13.26^abc^
CREAT (mg/dl)	1.96 ± 0.02	5.28 ± 0.14^a^	2.5 ± 0.02^ab^	2.32 ± 0.02^abc^
UREA (mg/dl)	33.70 ± 0.19	57.81 ± 0.47^a^	49.76 ± 0.520^ab^	39.01 ± 0.40^abc^

Each value is presented as the mean ± standard deviation (SD), n = 5 rats. The values are considered significant at p < 0.05, *C* normal control; *RA* Rheumatoid Arthritis; *M* Methotrexate; GHE, *G. mellonella* hemolymph. *CBC* complete blood picture, *WBCs* white blood cells count, *RBCs* red blood cells count, *Hb* hemoglobin, *PLTs* platelets. *ALT* Alanine transaminase, *AST* aspartate transferase, *ALP* alkaline phosphatase

^a^significance versus C; ^b^significance versus RA; ^c^significance versus RA + M

Compared with the control group, the RA + MTX group presented markedly lower (p < 0.001) PLTs and WBCs. Additionally, the Hb and RBC values were significantly lower (P < 0.05). In contrast, compared with the RA group, the RA + M group presented significant increases (p < 0.05) in Hb and RBCs.

The hemolymph treatment showed remarkable improvements when compared to the rheumatoid arthritis group. There was a substantial decrease (p < 0.001) in WBCs and a considerable increase (p < 0.001) in Hb and PLTs. Additionally, there was a significant increase (p < 0.05) in RBCs. Furthermore, the PLTs and WBCs were significantly greater (p < 0.0001) in RA + GHE-treated rats than in those treated with MTX.

Regarding the liver enzymes, Table 2 shows a highly significant increase (p < 0.001) in the serum ALT level in both the RA and RA + GHE groups compared with the control group. Moreover, the ALT level was significantly lower (p < 0.001) in the RA + GHE group than in the RA + MTX group.

Fortunately, serum AST levels exhibited a very significant increase (p < 0.001) among all studied groups, as compared to the control group. Moreover, RA + M and RA + GHE resulted in very significant reductions (p < 0.001) compared with those of the rheumatoid arthritis group, highlighting the preservation of the RA + GHE group, which presented the greatest reduction in values.

Similarly, the serum ALP levels were significantly elevated (p < 0.001) in all the experimental groups compared with those in the control group. Compared with the arthritis group, the MTX and hemolymph groups presented significantly lower ALP levels (p < 0.01 and p < 0.001, respectively). Additionally, compared with RA + MTX, *G. mellonella* hemolymph significantly decreased ALP levels (p < 0.05).

Compared with those in the control group, the serum creatinine and urea levels were significantly elevated (p < 0.001) in all the studied groups, whereas the MTX- and GHE-treated groups presented highly significant reductions (p < 0.001) compared with the RA groups. Compared with MTX-treated rats, GHE-treated rats presented highly significant reductions (p < 0.001) in creatinine and urea levels.

### Oxidative biomarkers

Compared with those in the control group, the serum levels of TAC in the different experimental groups were significantly lower (p < 0.001) than that of the control group (Table 3). However, compared with those in RA and RA + MTX groups, the mean values of TAC in the hemolymph in the experimental groups were significantly greater (p < 0.001).

**Table 3:** Oxidative and inflammatory mediators in different experimental groups. Data expressed as Mean ± SD

Groups	C	RA	AR + M	RA + GHE
Parameters				
Oxidative biomarkers				
TAC (mM/L)	1.142 ± 0.015	0.262 ± 0.014^a^	0.442 ± 0.012^ab^	0.489 ± 0.002^abc^
MDA (n M/g)	5.232 ± 0.189	8.768 ± 0.151^a^	7.69 ± 0.866^ab^	6.122 ± 0.21^abc^
SOD (U/g)	0.015 ± 0.0004	0.061 ± 0.0005^a^	0.057 ± 0.0005^ab^	0.048 ± 0.006^abc^
Inflammatory mediators				
CRP (pg/L)	12.78 ± 0.13	77.48 ± 0.78^a^	42.86 ± 1.25^ab^	18.94 ± 0.30^abc^
ACCP (ng/mL)	0.896 ± 0.07	5.14 ± 0.39^a^	2.56 ± 0.22^ab^	1.42 ± 0.22^abc^
IL-6 (PG/ML)	143.72 ± 1.57	325.16 ± 0.34^a^	183.72 ± 1.44^ab^	162.58 ± 0.54^abc^
TNF‐α (PG/ML)	31.16 ± 1.23	115.50 ± 0.91^a^	72.56 ± 0.71^ab^	43.30 ± 0.53^abc^
miR-146a	1.01 ± 0.01	5.56 ± 0.23^a^	2.50 ± 0.32^ab^	1.36 ± 0.18^abc^

Each value is presented as the mean ± standard deviation (SD), n = 5 rats. *C* normal control; *RA* Rheumatoid Arthritis; *M* Methotrexate; *GHE G. mellonella* hemolymph. *TAC* total antioxidant capacity in serum; *MDA* hepatic malondialdehyde; *SOD* Superoxide dismutase. *CRP* C—reactive protein; *ACCP* Anti-Cyclical Citrullinated Peptide Antibody; *IL-6* interleukin-6; *TNF‐α* tumor necrosis factor-alpha; *miR-146a* microRNA-146a. The mean difference is significant at *p* < 0.0

^a^significance versus C

^b^significance versus RA

^c^significance versus RA + M

Compared with those in the control group, hepatic MDA and SOD were significantly increased (p < 0.001) in all the experimental groups. However, hemolymph administration significantly reduced the hepatic levels of both MDA and SOD (p < 0.001) compared with those in the untreated and methotrexate-treated arthritis groups.

### Inflammatory mediators

The data in Table 3 revealed significant elevations (p < 0.001) in the serum levels of CRP and ACCP in the three arthritic groups, either untreated or treated, compared with those in the control group. Compared with RA and RA + MTX, *G. mellonella* hemolymph administration to arthritic rats significantly (p < 0.001) reduced the serum levels of CRP and ACCP.

The serum levels of IL-6 and TNF‐α, compared with those in the control group, were markedly significantly increased (p < 0.001) in all the studied groups. Moreover, the RA + MTX group presented highly significant reductions (p < 0.001) in the levels of IL-6 and TNF-α compared with those in the RA group. Additionally, the levels of both inflammatory mediators were markedly lower (p < 0.001) in the *G. mellonella* hemolymph-treated group than in the untreated and RA + MTX groups.

The data in Table 3 show a highly significant increase (p < 0.001) in the miR-146a expression level in the arthritic (RA) group compared with the normal control group. Although both the MTX- and *G. mellonella* hemolymph-treated groups presented highly statistically significant reductions (p < 0.001) in the miR-146a level, compared with those in the untreated arthritic group, the miR-146a level in the MTX-treated group was still significantly greater (p < 0.001 & p < 0.004) than that in the control group. Moreover, RA + GHE presented markedly lower (p < 0.001) miR-146a expression levels than RA + MTX-treated rats did.

**Fig. 5:**
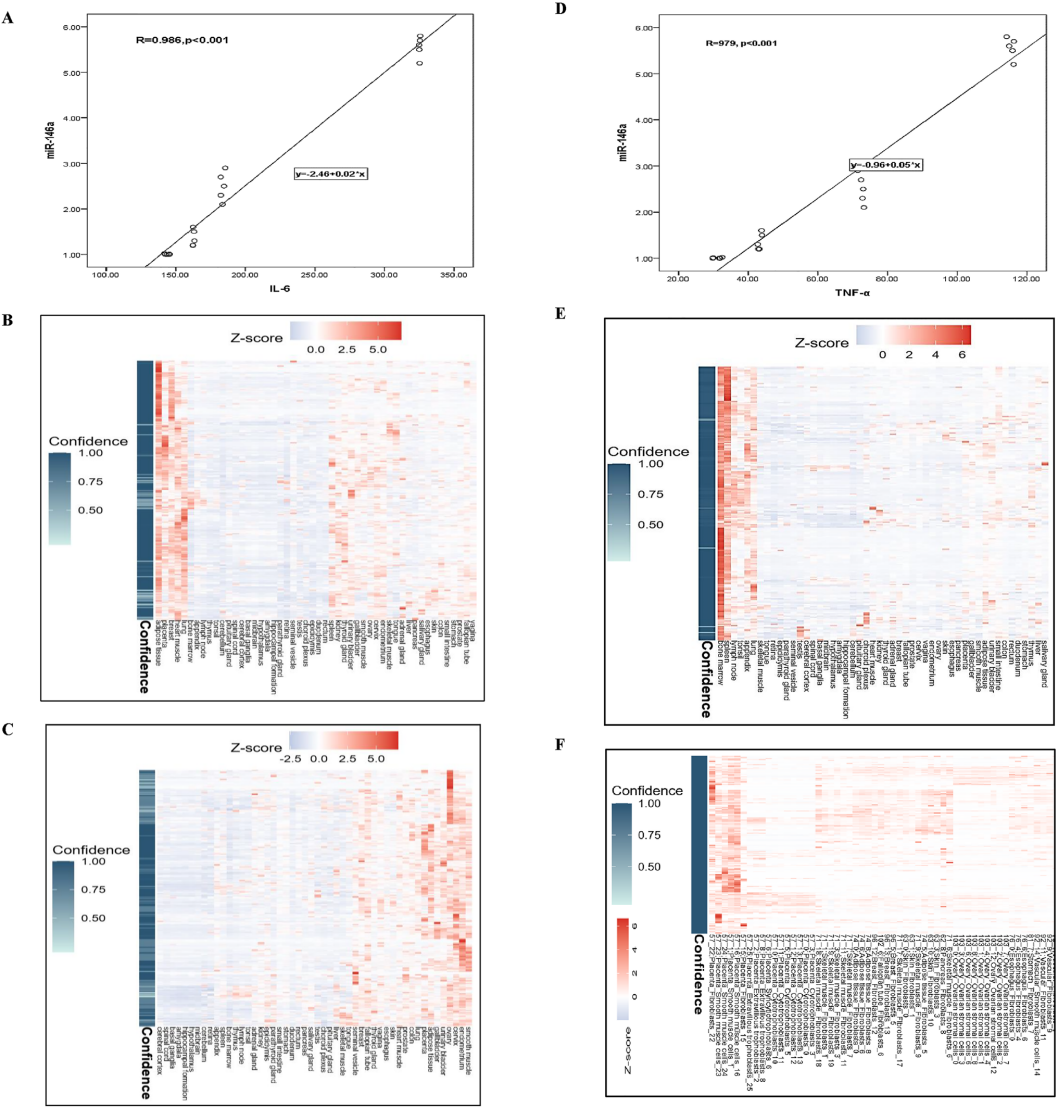
Correlation of miR-146a with IL-6 and TNF-α. **A** statistically significant positive correlation between miR-146a expression level and IL-6 (r = 0.982, p < 0.001). **B** IL-6 genes relative adipose tissue expression heatmap, a part of cluster 7 with confidence 1. **C** IL-6 genes relative connective tissue expression, a part of cluster 62 with confidence 1. **D** statistically significant positive correlation between miR-146a expression level and TNF-α (r = 0.978, p < 0.001). **E** TNF-α genes relative adipose tissue expression heatmap, a part of cluster 81 with confidence 1. **F** TNF-α genes relative connective tissue expression heatmap, a part of cluster 50 with confidence 1. N.B. relative expression from 0.0 to 1.0 (white to dark red color)

As shown in Figs. 5A & B, a significant positive correlation was observed between miR-146a and both IL-6 (r = 982, p < 0.001) and TNF-α (r = 978, p < 0.001).

### Histopathological and immunohistochemical

**Figs. 6:**
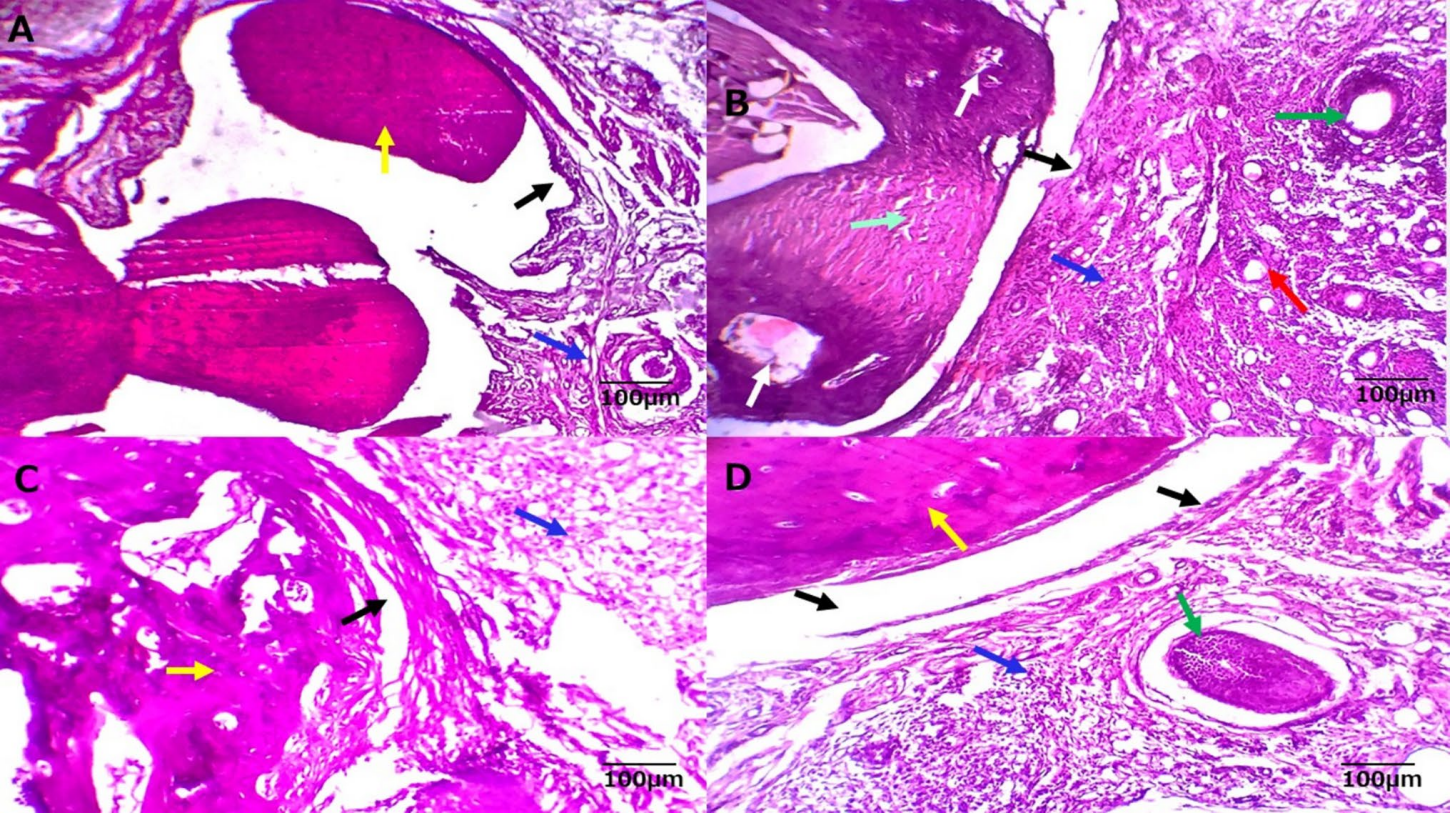
Histological manifestations from meta-tarsal joints of different experimental groups. **A** normal control group showing cortical bone having bone lamellae and osteocytes inside their lacunae (yellow arrows). The synovial membrane (black arrows), fibro hyaline joint capsule and the surrounding hypodermal tissue appear normal and free from any inflammatory or degenerative changes (blue arrows). **B** Arthritis group showing damage to all the joint compartments, surrounding tissues and represented by subchondral bone damage, and changes in the synovium that manifest as episodic synovitis. The bone tissue shows osteolytic degeneration, osteopenia and osteoporosis (light blue and white arrows), a marked inflammatory process is seen clearly in the synovial membrane and the adjacent articular capsule and tendon (tenosynovitis; black arrows), the predominant inflammatory cells are lymphocytes, plasma cells, macrophages and neutrophils, soft hypodermal tissue surrounding the affected joint shows aggregates of inflammatory cells (red arrows) with occasional adnexal damage and granulomatous nodular reaction (green arrows). **C** Methotrexate-Arthritis group showing remnants of cartilaginous and boney degenerative and necrotic changes (yellow arrows), associated with moderate inflammatory reaction in the synovial membrane (black arrows) and soft tissue (tenosynovitis; blue arrows). **D** Hemolymph-Arthritis group showing cartilaginous-bony regeneration with remarkable disappearance of the previous osteoporotic changes (yellow arrows), synovial membranous healing (black arrows) and resolving of most of inflammatory and/or granulomatous reactions in the surrounding soft hypodermal tissue with adnexal regeneration (blue and green arrows)

Histological analysis of H&E-stained sections from the metatarsal joint region in the control group revealed a thin cartilaginous cap composed of actively dividing chondrocytes, either singly or in pairs within lacunae (Fig. 6). This cap overlay a structurally normal cortical bone, characterized by bone lamellae and osteocytes within lacunae. Additionally, a smooth, continuous endosteal surface covered by osteoprogenitor and osteoblast cells was observed. The synovial membrane, fibrohyaline joint capsule, and surrounding hypodermal tissue appeared normal, without any inflammatory or degenerative changes. No exudative or transudative fluids were detected in bone marrow regions rich in hemopoietic cells (Fig. 6A).

In contrast, the arthritis group exhibited severe pathological changes involving the cartilage, bone, synovium, joint capsule, and surrounding hypodermal tissue, leading to widespread tissue damage. These alterations resulted in articular cartilage degradation, subchondral bone damage, bone marrow lesions, and episodic synovitis. Articular cartilage breakdown was characterized by chondrocyte degeneration and necrosis, occasionally forming cysts. The underlying bone displayed osteolytic degeneration, osteopenia, osteoporosis, and an increased presence of inflammatory cells infiltrating the interstitial tissue. Osteoclastic activity was also evident. A pronounced inflammatory response was observed in the synovial membrane and adjacent articular capsule and tendon (tenosynovitis), with predominant infiltration by lymphocytes, plasma cells, macrophages, and neutrophils. The surrounding hypodermal tissue also exhibited inflammatory cell aggregation, occasional adnexal damage, and granulomatous nodular reactions (Fig. 6B).

The treatment groups showed varying degrees of therapeutic efficacy. The hemolymph-treated group demonstrated the most significant improvements, with notable cartilaginous and bony regeneration, resolution of osteoporotic changes, and synovial membrane healing. Most inflammatory and granulomatous reactions in the surrounding soft tissue subsided, and adnexal regeneration was observed (Fig. 6D). In contrast, the MTX-treated group exhibited only partial improvement, with residual cartilage and bone degeneration, moderate inflammatory reactions in the synovial membrane and soft tissue (tenosynovitis), and limited healing in some cartilaginous and bony structures. Partial synovial membrane recovery was noted, but moderate inflammatory cell infiltration persisted in the bone marrow and surrounding soft tissue (Fig. 6C).

**Fig. 7:**
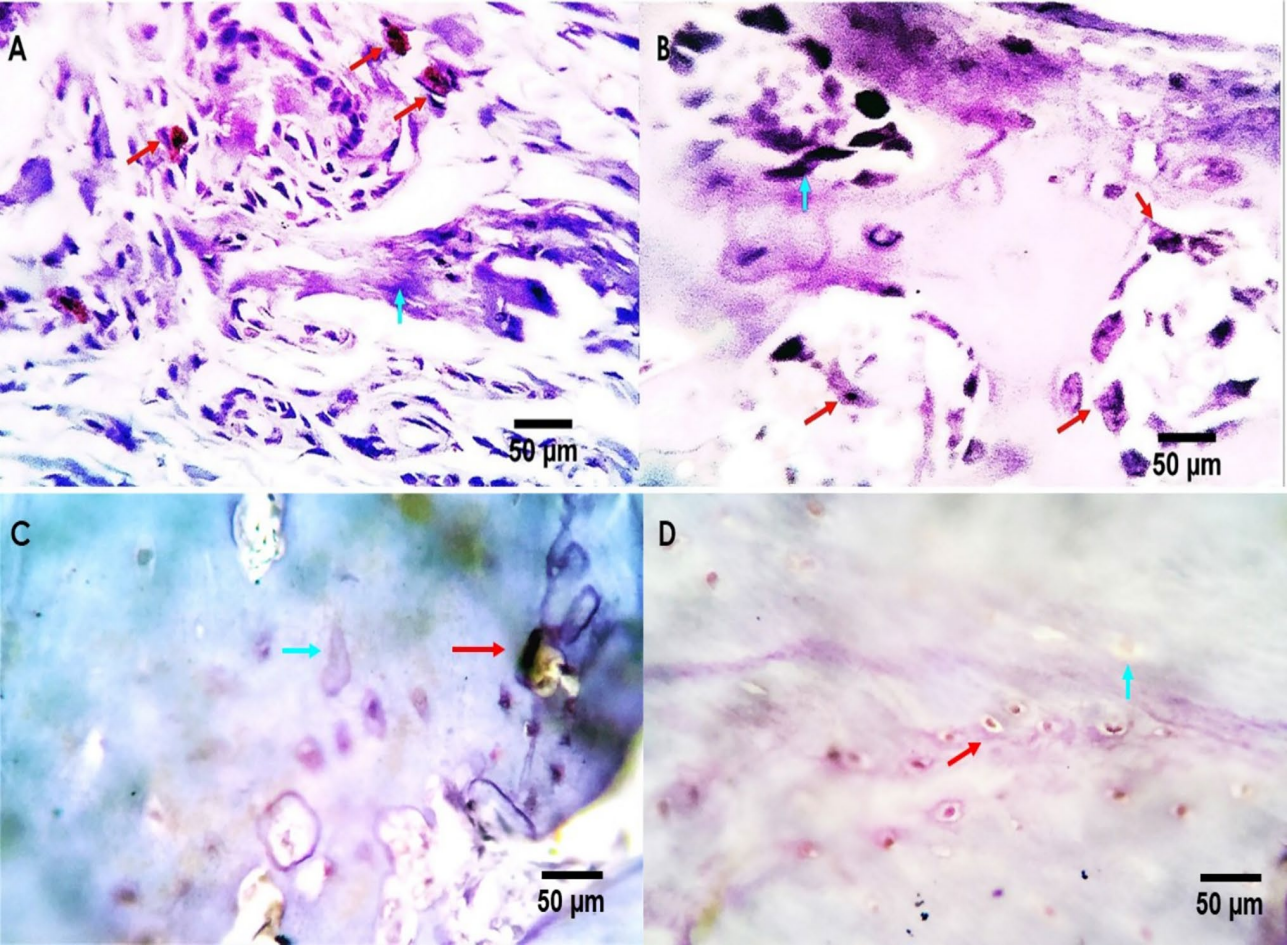
Anti-osteocalcin expression from metacarpal joint structures. **A** Control group showing a few reactive osteoblast cells in the peri-osteal and perichondral tissues cells. **B** Moderate to intense positive reactivity is seen involving both nuclear and cytoplasmic contents of a moderate number of bone osteoblastic and osteoclastic cells of the RA group (RA). **C** A mild to moderate number of osteoblasts and osteoclasts appears reactive to osteocalcin in methotrexate treated rats. **D** A few bone marrow monocytes and bone osteoblast are seen weakly expressed to osteocalcin in hemolymph treated rats. Positive cells are pointed by red arrows and negative cells are pointed by light blue arrows

OST protein expression was detected in arthritic chondrocytes, whereas both protein staining and specific mRNA expression were absent in the chondrocytes of normal adult cartilage (Fig. 7). In the control group, a few reactive osteoblasts were observed in the periosteal and perichondral tissues, as well as in bone osteoblastic cells (Fig. 7A). Positive reactivity was moderate to intense, involving both nuclear and cytoplasmic contents. In the arthritis group, a moderate number of osteoblasts and osteoclasts exhibited positive reactivity to the bone cell-stimulating marker OST (Fig. 7B).

In the RA + MTX group, a mild to moderate number of osteoblasts and osteoclasts showed reactivity to osteocalcin (Fig. 7C). Conversely, in the hemolymph-treated rats, only a few bone marrow monocytes and bone osteoblasts exhibited weak OST expression (Fig. 7D).

A morphometric analysis of osteocalcin-immunostained sections from the metacarpal joint structures of the control, RA, MTX-treated, and hemolymph-treated groups revealed positivity percentages of 6.75, 33.3, 16.78, and 5.81%, respectively.

## Discussion

Rheumatoid arthritis (RA) is an autoimmune disease characterized by joint swelling and deformity, which can lead to disability if not treated promptly [24]. Effective management of RA is challenging due to incomplete disease evaluation and medication side effects. Long-term reliance on a single drug may not be ideal, highlighting the need for natural therapies to modulate immunity and reduce inflammation. Biological treatments involve advanced RA management through targeting key inflammatory mediators [25]. Additionally, the increasing population of *G. mellonella* poses a growing economic threat, raising industrial and food maintenance costs. Innovative strategies are urgently needed to control this pest. Therefore, using larvae as a therapeutic agent with economic benefits, instead of harmful insecticides, may be advantageous. Hence, *G. mellonella* hemolymph was utilized in this study as an alternative eco-friendly therapy in the RA rat model.

The exploration of *G. mellonella* hemolymph spans computational molecular modeling through to in-vivo validation, providing a comprehensive approach to understanding its potential as a therapeutic agent. The objective of this study was to investigate the immune-modulatory impact of *G. mellonella* hemolymph as a potential anti-inflammatory and therapeutic antirheumatic natural agent. Specifically, the study focused on the crosstalk between miR-146a, IL-6, TNF-α, and osteocalcin in comparison with methotrexate (MTX) in adjuvant-induced arthritis (AIA), integrating computational molecular modeling and in-vivo validation.

HPLC analysis of *G. mellonella* hemolymph revealed compounds with notable antioxidant and anti-inflammatory properties. Quinic acid, known for scavenging free radicals, reduces oxidative stress and inflammation by inhibiting the TLR4-NF-κB and NF-κB-INOS-NO pathways [26]. Similarly, ellagic acid inhibits oxidative stress and proinflammatory cytokines [27]. Cinnamic acid has antioxidant and anti-inflammatory effects, improving oxidative and inflammatory parameters [28]. Chlorogenic acid alleviates oxidative stress and inflammation by inhibiting the HMGB1/TLR-4/NF-κB pathway and preventing mitochondria-mediated apoptosis [29]. It also regulates inflammatory mediators such as TNF-α and IL-6 [30]. Resorcinol decreases oxidative stress and inflammatory cytokine expression, enhancing therapeutic outcomes [31].

Computer-aided molecular design database predictions revealed complex interactions between CRP, OST, and PADI4, highlighting their roles in arthritis pathogenesis. CRP is associated with inflammation, OST with bone remodeling, and PADI4 with autoimmune responses through protein citrullination. These interactions, supported by in-vivo evaluations, underscore the importance of understanding their crosstalk in inflammatory pathways and bone metabolism. The bioinformatics analysis aligns with in-vivo assessments of cytokine levels and miR-146a regulation, reinforcing the molecular link between systemic inflammation and bone remodeling. This comprehensive approach provides valuable insights into RA pathophysiology and potential therapeutic targets.

Animal models of RA are commonly employed to test potential new therapies. The most frequently used models of human RA include adjuvant-induced arthritis (AIA) in rats and mice. The current animal model closely resembles human RA etiology. Measuring paw swelling in experimental models of arthritis provides insights into the effectiveness of interventions or potential therapeutic strategies. The current results revealed a significant increase in paw diameter in the rheumatoid arthritis group. Our results showed that the injection of 1 mg/ml CFA was related to substantial arthritis in the injected paw, consistent with findings from earlier studies [32, 33].

The administration of *G. mellonella* hemolymph in this study resulted in various physiological responses, including potential changes in body and relative organ weights. This may be due to the hemolymph’s bioactive compounds, which could modulate metabolism, oxidative stress [34], and inflammation [35]. The results suggest that hemolymph treatment does not significantly affect overall body weight, indicating no adverse effect on growth or nutritional status. Specific organs, particularly those involved in detoxification and immune responses, such as the liver, spleen, and kidneys, may show variations in weight, possibly due to the impact of hemolymph on oxidative stress levels, inflammatory processes, or immune modulation [36].

Inflammatory infiltration involves the migration and accumulation of immune cells into paw tissues, contributing to arthritis pathogenesis and swelling [37]. MTX and hemolymph administration significantly reduced paw edema, indicating their role in ameliorating RA progression.

MTX, a standard antirheumatic drug, has myelosuppressive effects, reducing Hb and RBC levels [38]. In contrast, hemolymph treatment increased Hb and RBC levels, likely due to its protective and regenerative effects. Its bioactive compounds may enhance erythropoiesis or shield RBCs from oxidative damage, which is common in RA. Additionally, hemolymph may mitigate MTX’s hematological side effects by supporting bone marrow and erythropoietic function [39].

The increase in WBCs indicates that *G. mellonella* hemolymph may modulate immune responses by regulating immune cell activation or cytokine release [40]. The decrease in PLT count suggests its antioxidative effects, as platelets play a role in inflammation and oxidative stress. This reduction implies that hemolymph may exert its benefits by modulating oxidative stress-related pathways [41].

Compared to MTX treatment, GHE eliminates associated adverse effects and offers protective effects on liver and kidney function, likely due to its ability to scavenge free radicals and mitigate oxidative damage [36]. Hemolymph’s ability to restore oxidative balance by increasing total antioxidant capacity may be due to its bioactive molecules with antioxidant properties [34]. Natural antioxidants demonstrate their qualities by scavenging radicals, preventing lipid peroxidation, and protecting structural proteins [42].

GHE treatment significantly reduced proinflammatory IL-6 and TNF-α levels compared to RA and RA + MTX groups, highlighting its enhanced anti-inflammatory activity due to phenolic compounds identified through HPLC analysis [43]. Hemolymph modulates inflammatory mediators without adversely affecting immune system organs. IL-6, TNF-α, and PADI4 are highly expressed in monocytes/macrophages, promoting inflammation and osteoclast-driven bone resorption, while BGLAP (osteocalcin) is selectively expressed in osteoblasts/osteocytes, indicating its role in bone matrix production [35, 44]. Hemolymph treatment may mitigate inflammation more effectively than MTX, potentially influencing autoantibody production and attenuating the autoimmune response associated with RA [36, 45].

MiR-146a is a negative regulator of the inflammatory response, with its overexpression reflecting the interplay between proinflammatory and anti-inflammatory mechanisms in arthritis [46]. Bioactive compounds in *G. mellonella* hemolymph extract contribute to its anti-inflammatory and immunomodulatory properties, evidenced by the downregulation of miR-146a expression in treated rats compared to untreated and RA + MTX-treated rats. These findings align with those of Sadek et al. [43] and Mashaal et al. [47], who demonstrated that hemolymph extract effectively reduces inflammation. By lowering the levels of proinflammatory mediators such as IL-6 and TNF-α, hemolymph extract as an anti-inflammatory treatment may also lead to a reduction in miR-146a levels, which are associated with inflammatory stimuli.

Histopathological analysis of RA rat joints confirmed severe inflammation, with lymphocytes, plasma cells, macrophages, and neutrophils infiltrating the surrounding tissue. These proinflammatory cells contribute to cartilage degradation and joint damage by activating matrix metalloproteinases [48]. The correlations among miR-146a, IL-6, TNF-α, and OST demonstrated that miR-146a reduces inflammation by negatively regulating IL-6 and TNF-α. This highlights a critical balance where inflammation control through TLR4 inhibition [49] and miR-146a regulation could either preserve or disrupt bone remodeling in RA [50].

Rheumatoid arthritis increases bone resorption due to disrupted osteoclastic-osteoblastic activity [51]. Immunohistochemical analysis revealed OST expressions in the RA- and MTX-treated groups, whereas the normal and hemolymph-treated groups showed reduced OST expressions, suggesting a protective effect. These findings indicate that RA induces chondrocyte hypertrophy, which is linked to disease severity.

## Conclusions

In conclusion, this study reports interesting findings that *G. mellonella* larval hemolymph improves the inflammatory state by modulating the crosstalk among miR146a, IL-6, TNF-α, and osteocalcin. It achieves this by reducing the production of inflammatory mediators and acting as an antioxidant agent, thereby modulating arthritic pathogenicity. However, further studies are recommended to comprehensively understand the underlying signaling mechanism of these impacts.

## Supplementary Information

Below is the link to the electronic supplementary material.

Supplementary file1 (DOCX 15 KB)

## Data Availability

Data will be available under reasonable request from the authors.

## Abbreviations

ACCP: Anti-Cyclical Citrullinated Peptide Antibody
AIA: Adjuvant induced-arthritis
CRP: C-reactive protein
DMARDs: Disease-modifying antirheumatic medications
*G. mellonella*: *Galleria mellonella*
GHE: *G. Mellonella* hemolymph extract
MDA: Malondialdehyde lipid peroxidation
MTX: Methotrexate
NSAIDs: Non-steroidal anti-inflammatory medicines
RA: Rheumatoid arthritis
TAC: Total antioxidant capacity
TRAF6: Tumor necrosis factor receptor-associated factor 6
