# Supplementary material for: MiR-146/TNF-α/IL-6/osteocalcin crosstalk in anti-rheumatic potential of Galleria mellonella hemolymph from computational molecular modeling to in-vivo validation
Source: J Comput Aided Mol Des. 2025 Apr 23;39(1):17. doi: 10.1007/s10822-025-00595-3 (PMC12018512; doi:10.1007/s10822-025-00595-3)
Supplement: Supplementary file 1 — Supplementary file1 (DOCX 15 KB) [file 10822_2025_595_MOESM1_ESM.docx]

list of previously published studies that aimed the usage of hemolymph extracted from insects and discuss its Immunomodulatory potential in in-vivo infection rat model.

1. <https://doi.org/10.47278/journal.ijvs/2022.192>
2. <https://doi.org/10.1038%2Fs41598-024-57113-y>
3. [www.doi.org/10.14202/vetworld.2020.1599-1604](http://www.doi.org/10.14202/vetworld.2020.1599-1604)

The previous technically sound in vitro work for hemolymph application.

[www.doi.org/10.14202/vetworld.2020.1599-1604](http://www.doi.org/10.14202/vetworld.2020.1599-1604)
